# Intracranial Hemorrhage Complicating Herpes Simplex Encephalitis on Antiviral Therapy: A Case Report and Review of the Literature

**DOI:** 10.1155/2017/6038146

**Published:** 2017-09-19

**Authors:** Ghada ElShimy, Christina Mariyam Joy, Fred Berlin, Waleed Lashin

**Affiliations:** ^1^Department of Internal Medicine, St. Joseph's Regional Medical Center, New York Medical College, Paterson, NJ, USA; ^2^Department of Diagnostic and Interventional Radiology, St. Joseph's Regional Medical Center, New York Medical College, Paterson, NJ, USA

## Abstract

Herpes simplex virus (HSV) encephalitis is the most common cause of nonendemic sporadic encephalitis in the USA. Decreased mortality with early treatment with acyclovir has been documented. Although common complications include cortical petechial hemorrhages, frank intracerebral hematomas are considered very rare. Only few cases have been reported in the literature. We report a case of HSV encephalitis complicated by intracerebral hemorrhage 12 days after initiation of acyclovir therapy.

## 1. Introduction

Herpes simplex virus encephalitis is the most common cause of nonendemic sporadic encephalitis in the USA. Mortality can be as high as 70% when left untreated [1, 2]. Early initiation of treatment with acyclovir is associated with reduced mortality. However some neurological sequela often persist in these patients. Frank hematoma is extremely rare despite initiation of treatment; only 27 cases have been reported in the literature [2–20].

## 2. Case Report

A 49-year-old male with past medical history of type-1 diabetes mellitus, hepatitis B, chronic hepatitis C, intravenous drug abuse, and traumatic brain injury sustained after fall 2 years prior to presentation (with left hemisphere encephalomalacia) with no history of gastrointestinal bleed (not on aspirin or any anticoagulation treatment at home) presented to the emergency department (ED) with headache, altered mental status, and fever for 2 days' duration.

**Figure 1:**
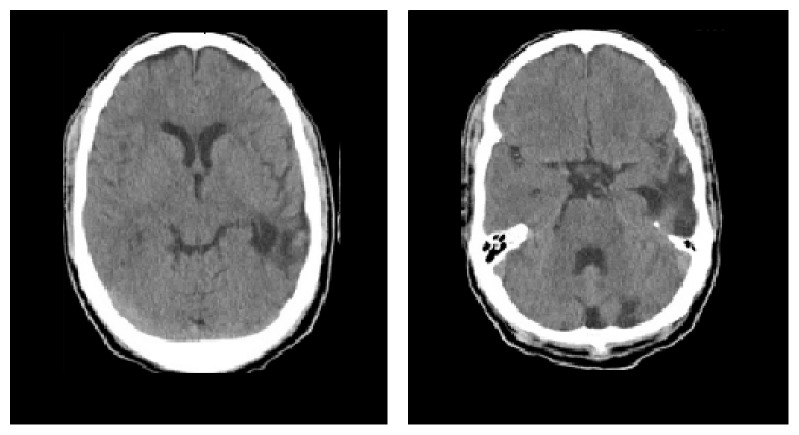
(Day 1) noncontrast CT head showing encephalomalacia on the left hemisphere without hemorrhage.

On arrival, patient was febrile with temperature of 102 F, pulse rate of 112 beats per minute, blood pressure of 143/80 mmHg, and respiratory rate of 20 breaths/minute with saturation of 100% on room air. Physical exam revealed a well-built and nourished gentleman, awake and orientated to person and place with Glasgow coma scale of 15. Fundoscopic exam revealed no papilledema. He was able to move all four extremities but had positive meningeal signs. Laboratory tests showed white blood cell (WBC) count of 9100/mm^3^ (72% polymorph nuclear (PMN) leukocytes and 19% lymphocytes). Patient had a lumbar puncture done and cerebrospinal fluid (CSF) analysis showed WBC count of 45 cells/mL (72% PMN leukocytes and 24% lymphocytes); protein of 78 mg/dL; and glucose of 201 mg/dl. Noncontrast computed tomography (CT) head showed an old left hemisphere encephalomalacia without any acute intracranial pathology (Figure 1).

**Figure 2:**
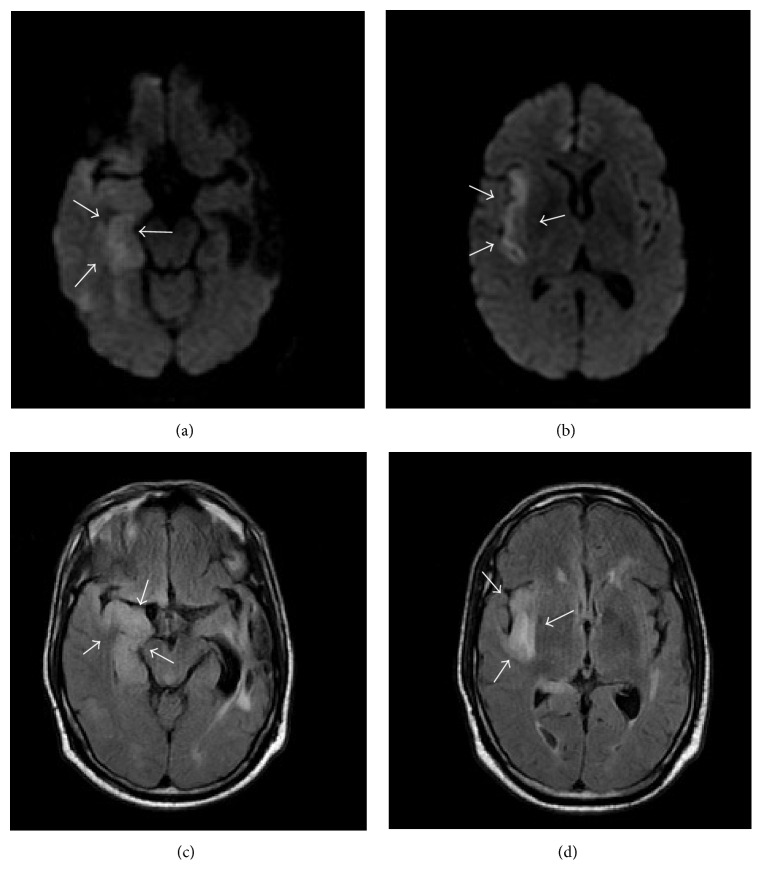
(Day 2) ((a) and (b)) noncontrast MRI of the brain: Diffusion Weighted Imaging (DWI) demonstrates increased signal in the right temporal lobe (a) and right insula (b), consistent with cytotoxic edema. Restricted diffusion is less intense as compared to infarction. These imaging findings in combination with clinical scenario are highly suggestive of herpes simplex encephalitis. ((c) and (d)) Noncontrast MRI of the brain: FLAIR sequence demonstrates asymmetrical involvement of the right medial temporal lobe (c) and the insular cortex (d). Unlike in middle cerebral territory infarct, basal ganglia is spared. Arrows in (a) refer to increased signal in right temporal lobe, in (b) increased signal in right insula, in (c) FLAIR sequence demonstrating involvement of right medial temporal lobe, and in (d) FLAIR sequence demonstrating involvement of right insular cortex.

Patient was treated empirically for meningoencephalitis (pending cultures) with vancomycin, ceftriaxone, and acyclovir plus first dose of dexamethasone prior to initiation of antibiotics and droplet precaution. Subsequently noncontrast magnetic resonance imaging (MRI) of the brain showed right temporal lobe involvement suggestive of HSV encephalitis (Figure 2). Electroencephalography (EEG) showed slowing in the right frontoparietal area with no electrographic seizures. Patient's CSF cultures came back negative for both bacteria and HSV deoxyribonucleic acid (DNA) by polymerase chain reaction. But based on the patient's initial clinical presentation, EEG and MRI highly suggestive of HSV encephalitis, antibiotics were discontinued and acyclovir was continued. In the next days, fever subsided and neurological status improved so patient was discharged home on acyclovir treatment.

**Figure 3:**
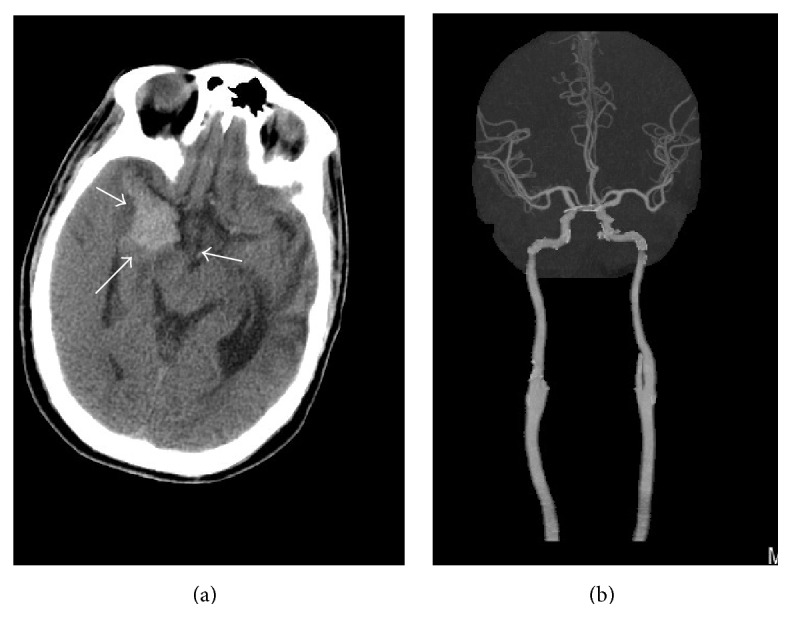
(Day 12) (a) noncontrast CT of the brain demonstrates interval development of acute hemorrhage within medial right temporal lobe, a rare complication of herpes simplex encephalitis. (b) CT angiogram with IV contrast: coronal MIP reconstruction demonstrates patency of the intracranial circulation, with widely patent intracranial arteries, including middle cerebral arteries. Arrows in (a) refer to area of acute hemorrhage within the right medial temporal lobe.

Twelve days from the initial presentation, patient was brought back to the ED due to acute worsening of mental status. There was no trauma or fall as per family. He was confused, alert but disoriented to person, time, and place, and not following commands. Physical exam did not show any signs of trauma. Laboratory tests showed platelets count 343 × 10^3^/mm^3^, partial thromboplastin time (PTT) 29.1 seconds, prothrombin time (PT) 14.3 seconds, and international normalized ratio (INR) 1.1. Noncontrast CT of the brain demonstrated interval development of acute hemorrhage within medial right temporal lobe, a rare complication of herpes simplex encephalitis. Computed tomography angiography (CTA) done at the same time was negative for any underlying vascular lesion (Figure 3). Neurosurgery team was consulted and patient was treated conservatively. Subsequent CT scans done showed gradual improvement of the hematoma. Patient was continued on IV acyclovir and was discharged. Patient was followed up over the next 2 years and he returned to his baseline neurological status.

## 3. Discussion

Herpes simplex virus encephalitis usually has 2 peaks in incidence (at ages of 20s and 50s). There is no seasonal variation. Risk is not increased in immunocompromised patients; however it is more documented in HIV positive individuals. Our patient had diabetes mellitus, hepatitis B, and hepatitis C but not HIV. HSV type I occurs in vast majority of patients; however few cases can occur with HSV-2. The disease is fatal with very high mortality rate in untreated individual (70% mortality) with no regain of full neurological function except in 2.5% of these patients [1, 21, 22].

The decision to initiate antiretroviral therapy in a patient with suspected viral encephalitis is often clinical. Although serological testing is of prognostic value for identification of specific pathogens, it should not delay the treatment. Detection of antibodies of HSV virus in the CSF may not provide adequate yield especially if the sample is collected early during the convalescent stage of the disease as what occurred in our patient. Hence subsequent testing is helpful [23, 24]. Neurodiagnostic evaluation can provide support for the diagnosis by the demonstration of temporal lobe edema or hemorrhage by magnetic resonance image scan as well as spike and slow-wave activity on electroencephalogram [25]. In our patient, initial HSV PCR was negative; however the clinical scenario, CSF results, MRI, and EEG, all of them, supported the diagnosis of HSV encephalitis especially in the setting of clinical and neurological improvement with acyclovir treatment and resolution of fever in the next days.

In literature, it was documented that MRI is more significant in early detection of HSV encephalitis than CT scan with better sensitivity [26]. Around 90% of MRI done within the first 48 hours of admission are abnormal in HSV encephalitis. Early finding on MRI usually occurs in the cingulate gyrus and medical temporal lobe. They are characterized by gyral edema on T1 weighted images and high signal intensity on T2-weighted and T2 FLAIR (87.7% specificity) [27–29]. Our patient had right medial temporal lobe lesion characteristic of HSV encephalitis.

Other invasive diagnostic tests include brain biopsy which is considered the most sensitive and specific diagnostic tool. However, it is reserved for patients who does not respond to acyclovir therapy and if the etiology cannot be confirmed by CT or MRI that is why it was not required in our patient. There are no specific EEG patterns that are pathognomonic for HSV encephalitis. However some features are helpful in the diagnosis as focal or lateralized EEG abnormalities [30]. Our patient had slowing in right frontoparietal region in the presence of encephalitis which also supported the diagnosis.

Intracranial hemorrhage is a rare complication of herpes encephalitis despite early initiation of antiviral therapy. Necrotizing infiltrate with scattered foci of small hemorrhage in the brain is usually seen; however, frank hematoma is rare and usually occurs during the second week after initial presentation. Intracranial pressure continues to rise and peaks at 11-12 days from onset of illness possibly explaining why our patient developed frank hematoma on day 12. However, there are other reported cases when bleeding occurred earlier [31]. Our patient had normal platelet count with normal coagulation profile, absent aneurysm, or any vascular abnormalities on CTA and he was not on aspirin or anticoagulation therapy excluding other causes of intracranial hemorrhage. Interestingly the intracerebral hemorrhage was just localized to the area of prior HSV presented 12 days earlier.

The exact mechanism for hemorrhage is unclear. The hemorrhage was proposed to be likely due to small vessel vasculitis resulting in endothelial damage with secondary bleeding. This was explained based on the finding of fibrinoid necrosis in the pathological samples of evacuated hematoma [7, 13]. Some theories stated that bleeding can be due to rupture and transient hypertension caused by raised intracranial pressure [31]. Another hypothesis includes immune mediated inflammatory reaction that would damage the brain tissue and make it vulnerable to bleeding [13]. The occurrence of the hemorrhage at the site of an abnormal MRI signal suggests that changes in the brain parenchyma and vessels induced by the encephalitis carry a potential risk of spontaneous bleeding [7], so close monitoring of neurological status is recommended in these patients. Fortunately, our patient's neurological status improved to his baseline.

## 4. Conclusion

Intracranial bleeding, although infrequent, is a possible complication of HSV encephalitis even in the setting of early treatment with antiviral. Neurological exam follow-up is essential in these patients. Early imaging studies are required when there is decline in neurological status and/or signs of increased intracranial pressure to rule out intracranial hemorrhage.

## Abbreviations

HSV: Herpes simplex virus
CT: Computed tomography
MRI: Magnetic resonance imaging
FLAIR: Fluid attenuated fluid recovery
DWI: Diffusion Weighted Imaging
MIP: Maximum intensity projection
EEG: Electroencephalography
DNA: Deoxyribonucleic acid
WBC: White blood cells
PMN: Polymorph nuclear
ED: Emergency department
CSF: Cerebrospinal fluid
PTT: Partial thromboplastin time
PT: Prothrombin time
INR: International normalized ratio.
